# Alcohol Mixed with Energy Drinks: Consumption Patterns and Motivations for Use in U.S. College Students

**DOI:** 10.3390/ijerph8083232

**Published:** 2011-08-05

**Authors:** Cecile A. Marczinski

**Affiliations:** Department of Psychological Science, Northern Kentucky University, Highland Heights, KY 41042, USA; E-Mail: marczinskc1@nku.edu; Tel.: +1-859-572-1437; Fax: +1-859-572-6085

**Keywords:** alcohol, energy drinks, college students, binge, motivation for use

## Abstract

Binge drinking in college students is widespread and known to cause significant harms and health hazards for the drinker. One factor that may be exacerbating hazardous drinking in young people is the new popular trend of consuming alcohol mixed with energy drinks (AmED). However, rates of AmED use and motivations for AmED consumption in college students have not been well established. In this study, 706 undergraduate college students from a university in the United States participated in a web-based survey that queried self-reported alcohol, energy drink, and AmED use. In addition, motivations for using AmEDs were assessed. The results indicated that for all participants, 81% reported that they have tried at least one energy drink in the past and 36% reported consumption of at least one energy drink in the past 2 weeks. Alcohol consumption patterns were similar to findings from U.S. national surveys of college drinking, as 37% of respondents were classified as binge drinkers and 23% abstained from drinking. In the whole sample (including the alcohol abstainers), 44% reported trying AmED at least once and 9% reported AmED consumption at least once in the past 2 weeks. 78% of respondents agreed with the statement that AmEDs appeal to underage drinkers. When AmED users were asked about various motivations for consuming AmEDs, users reported that they consumed these beverages to get drunk and reduce sedation compared to alcohol alone. In conclusion, the consumption of AmEDs is common in U.S. college students. Motivations for using AmEDs include the reduction of the sedative effects of alcohol, an important interoceptive cue that one should stop drinking.

## 1. Introduction

Underage drinking and binge drinking among college students is widespread and known to cause significant harms and hazards for the drinker and those around the drinker [1–3]. Heavy episodic (binge) drinking has been argued to the number one public health hazard and the primary source of preventable morbidity and mortality for the more than six million college students in the United States [4]. Epidemiological evidence has shown that binge drinking is widespread on U.S. college campuses, with almost half of students reporting binge drinking. In addition, binge drinking has been associated with unplanned and unsafe sexual activity, assaults, falls, injuries, criminal violations, and automobile crashes [5–7]. Binge drinkers are 14 times more likely to drive while impaired by alcohol than are nonbinge drinkers [8] and driving while intoxicated is more directly associated with binge drinking than with chronic heavy drinking [9,10]. Approximately half a million college students in U.S. are injured and 1,700 die each year from alcohol-related injuries [11]. Worldwide, 1.8 million deaths annually are the result of injuries cause by hazardous and harmful drinking, accounting for 3.2% of all deaths and 4.0% of disease burden [12].

In the year 2000, the U.S. Surgeon General established a 50% reduction in college binge drinking by the year 2010 as one of its health goals for the United States [13]. Despite various significant efforts to change this health risk behavior, current levels of binge drinking in young people in the U.S. appear to be relatively unchanged from year 2000 levels [14,15]. Thus, the constancy of underage and binge drinking behavior in young people, despite increased attention to the problem, begs the question of what unexamined factors may be contributing to the binge drinking problem. One possible variable that has received extremely little research attention thus far and is the focus of this article is the shift in alcoholic drink preferences in high school and college students in the past decade. Young people have become enamored with the trend of mixing of energy drinks with alcohol (e.g., Red Bull and vodka or other caffeinated cocktails like Jagerbombs, which are a mix of Jagermeister and Red Bull) [16–22]. However, little is known about how commonly these beverages are consumed in the college student population and whether the motivations associated with consumption of these drinks contribute to hazardous drinking.

Energy drinks (e.g., Red Bull, Monster and Rockstar) are beverages marketed with claims of providing users with increased alertness and energy boosts [18]. These beverages contain a variety of compounds including plant-based stimulants (e.g., guarana), simple sugars (e.g., glucose, fructose), amino acids (e.g., taurine) and herbs (e.g., ginseng) [19]. However, most researchers agree that that the extremely high caffeine content (the principal active ingredient) of these beverages drives the stimulant properties that users often report following consumption [21,23]. The U.S. Food and Drug Administration (FDA) does not regulate the caffeine content of energy drinks and the caffeine content of these beverages can contain 150%–300% of the amount of caffeine that the FDA allows for cola beverages [17].

Despite the exponential rise in sales in the U.S. and worldwide energy drink market [24], little research has examined the rates of use and motivations for consumption of energy drinks alone and mixed with alcohol in college students. The limited survey data from college students does reveal that the consumption of energy drinks, alone and in combination with alcohol, seems to be common. Malinauskas and colleagues used a 19 item survey to assess energy drink consumption patterns in 496 college students at a state university in the central Atlantic region of the United States [25]. The authors reported that 51% of participants reported the consumption of at least one energy drink per month. Many of the self-reported energy drinks users consumed three or more energy drinks at a time when combining them with alcohol while partying. Similar findings were reported by O’Brien and colleagues who used a web-based survey to examine consumption patterns in 697 college students [19]. The authors reported that 24% of past 30-day alcohol drinkers reported consuming alcohol mixed with energy drinks (AmED) in the past 30 days. Moreover, students who reported AmED consumption reported significantly higher alcohol-related consequences, such as riding with an intoxicated driver, being physically hurt or injured, and requiring medical treatment, even after adjusting for the amount of alcohol consumed. Similarly, a survey of 602 undergraduate students found that frequency of energy drink consumption was positively associated with a variety of health risk behaviors, such as marijuana use, sexual risk-taking, fighting, and not wearing a seatbelt when riding in a car [18].

Another important limitation to these previous studies is that they did not report typical alcohol consumption patterns for their sample and compare them to national college rates of alcohol use. Typical alcohol use is an important reference point in a survey of AmED use in a college sample since U.S. colleges and universities can differ dramatically in typical alcohol consumption patterns, ranging from almost no students who binge drink to more than 70% of students reporting past 2 week binge drinking [3,26]. Thus, it is unclear from data from previous surveys of AmED use if the participants were typical of the majority of college students in the U.S. in their alcohol consumption patterns. Several national surveys of college students have noted the largely stable pattern of alcohol use on college campuses in the U.S. As one example, the Harvard School of Public Health College Alcohol Surveys have revealed that binge drinking is a widespread problem on college campuses with almost half of the students reporting binge drinking and 1/5 students reporting abstaining from alcohol consumption. However, more moderate to limited alcohol use is observed at religious schools, commuter schools and historically black colleges and universities [3–6].

It is unclear why young drinkers are motivated to consume AmEDs. Preliminary research suggests that motivations important for AmED consumption include to consume the drink while partying and to improve the taste of alcohol [19,25,27,28]. Improved knowledge about motivations for AmED consumption is critical in light of findings from three recent studies that highlight the risks of consuming these drinks. Price and colleagues reported that, relative to alcohol drinking episodes in which energy drinks were not used, participants self-reported drinking significantly more alcohol when using AmEDs [20]. In addition, results from a field study of college student patrons leaving local bars revealed that patrons who had consumed AmEDs were at a 3-fold increased risk of leaving the bar highly intoxicated and a 4-fold increased risk of intending to drive home, compared to other drinking patrons [22]. Finally, results from a recent laboratory study where subjects were blind to beverages that administered revealed that subjects who received an AmED beverage reported feeling more stimulated compared to subjects who received alcohol alone. However, both AmED and alcohol subjects were impaired on an impulse control task, compared to subjects who did not ingest alcohol [17]. Therefore, findings from these three studies are worrisome and indicate that AmEDs may lead to more hazardous drinking.

What is not known is if drinkers of AmEDs are unaware of how the stimulant effects of energy drinks with alcohol reduce sedation associated with drunkenness, or if drinkers are motivated to consume AmEDs because they are aware that the stimulant effects of energy drinks with alcohol can lead to greater drinking and longer drinking episodes. Asking drinkers about their motivations for using AmEDs could help in efforts to disentangle whether the risks that coincide with consuming these drinks are intentional or unintentional. At present, the paucity of knowledge about motivations for using energy drinks with alcohol is problematic for adequately informing the public about the risks of these drinks. Having limited knowledge about the motivations of young people for using these energy drink beverages with alcohol makes it difficult to judge if these beverages are contributing to hazardous binge drinking in young people.

Therefore, the purpose of this study was to collect anonymous survey responses from college students about their alcohol and energy drink consumption habits and their motivations for consuming alcohol mixed with energy drinks. Questions from previously published questionnaires (e.g., the Harvard School of Public Health College Alcohol Survey) were used to survey alcohol use habits. Additional questions were added to further query respondents about typical energy drink usage and the mixing of energy drinks and alcohol. Participants were also asked about a variety of possible motivations for using AmEDs.

## 2. Materials and Methods

### 2.1. Study Population

Seven hundred and six (706) psychology undergraduate students (354 males) at Northern Kentucky University (NKU) completed an anonymous Internet-based online survey of beverage consumption patterns. NKU (Highland Heights, KY, USA) is a 4-year public university with an undergraduate enrollment of approximately 12,000 students. 1,210 students from the psychology department were eligible to participate in this survey during the fall semester, and 706 were recruited, resulting in a 58% response rate. Note that students were offered multiple choices of research studies to participate in, to limit the coercive aspect of recruiting. Informed consent was obtained from all participants in accordance with the university’s institutional review board for the protection of human subjects. Participants received partial course credit for participation in psychology research for completing the survey. Data collection occurred during September, October and November of 2008.

### 2.2. Apparatus and Materials

The use of a web-based survey ensured the complete anonymity of responses and that responses of participants could not be tied to personal information including the name or course instructor or any other identifying feature of the participant. Given that the legal drinking age is 21 years in the U.S., anonymous data collection afforded us greater ability to recruit more subjects without participants having concerns about the legal ramifications of admitting to their illegal alcohol consumption activities. Participants were informed that the survey contained 188 questions and would take approximately 45 minutes to complete. The questions queried demographic information (age, gender and race), energy drink consumption patterns (rates of use), alcohol consumption patterns, consumption patterns of alcohol mixed with energy drinks (AmED) and motivations for using AmEDs. The demographic and alcohol use questions were similar to those used in Harvard College Alcohol Surveys (CAS) [27]. These questions ask the participant about drinking behavior in the past 2 weeks. For example, the participant would be asked, “Think back over the last 2 weeks. How many times did you have 5 or more drinks in a row?”. The energy drink and AmED questions were modified based on the CAS survey questions. Additional questions were developed after reviewing the available published surveys regarding energy drink and AmED use studies [18,19,25]. A small pilot study at the university revealed additional motivations that should be included for AmED use. Some questions had yes/no responses (e.g., Have you consumed an energy drink in the past 2 weeks?). Other questions pertaining to motivations for use utilized a 4-point Likert-type scale with response options of 1 (strongly disagree), 2 (disagree), 3 (agree) and 4 (strongly agree).

Upon completion of data collection, participants were classified as binge and non-binge drinkers based on one widely-used definition of a binge-drinking episode as drinking five or more drinks on an occasion in the past 2 weeks for men and four or more drinks on an occasion in the past 2 weeks for women [2–6].

### 2.3. Data Analysis

The goals of data analysis were to: (1) report prevalence estimates of energy drink consumption, alcohol consumption, and AmED consumption; and (2) examine the motivations for using AmEDs in the regular users of these beverages. For motivations for use, one-sample t-tests (2-tailed) were utilized to test against the null value of 2.5, since those items were responded to on a 4-point Likert scale with 2.5 as the midpoint between the ratings of 2 (disagree) and 3 (agree). All analyses were performed using SPSS v.17.0 and the alpha value was set at .05.

## 3. Results and Discussion

### 3.1. Participant Demographics

Seven hundred and six participants (354 males) with a mean (SD) age of 20.9 (5.3) years participated in this study. The self-reported racial make-up of the sample was 88.7% Caucasian, 5.5% African-American, 1.7% Hispanic/Latino, 1.4% Asian American, and 2.7% other (which included American Indian, Alaska Native, Native Hawaiian, Pacific Islander, and multiracial). Participants identified his or her class rank as freshman (58.4%), sophomore (17.3%), junior (13.0%), senior (10.1%), or other (1.3%, which included continuing education or graduate study). Greek affiliation was queried with 8.9% reporting membership in a fraternity or sorority.

### 3.2. Prevalence Estimates of Energy Drink Consumption

Table 1 displays the prevalence estimates for energy drink consumption for all respondents. The table illustrates that the majority of respondents (81.4%) have tried an energy drink at least once in the past with only 18.6% of the sample have never tried an energy drink. Moreover, 36.4% of the entire sample described themselves as current consumers of energy drinks (*i.e.*, the participant reported consuming at least one energy drink in the past two weeks).

### 3.3. Prevalence Estimates of Alcohol Consumption

Table 2 displays the alcohol consumption patterns for all respondents. The table illustrates that 43.5% did not consume alcohol in the past 2 weeks whereas 37.0% met the criteria of binge drinking. Binge drinking was defined as the consumption of five or more drinks on one occasion on one or more days during the past 2 weeks for males. For females, binge drinking was defined as the consumption of 4 or more drinks on one occasion on 1 or more days during the past 2 weeks. Individuals who reported consumption of at least one drink of alcohol in the past 2 weeks but did not meet the criteria for binge drinking were labeled as current drinkers with no binge drinking (19.5% of the sample).

### 3.4. Prevalence Estimates of Alcohol Mixed with Energy Drink Consumption

Table 3 illustrates the prevalence estimates for alcohol mixed with energy drinks (AmED) consumption for all survey respondents (including those individuals who did not drink any alcohol in the past 2 weeks). For the entire sample, 44.0% had tried AmEDs or were regular consumers of AmEDs. Recent use of AmEDs (in the past 2 weeks) was reported by 9.3% of the entire sample.

### 3.5. Motivations for Alcohol Mixed with Energy Drinks Consumption

**Figure 1.**
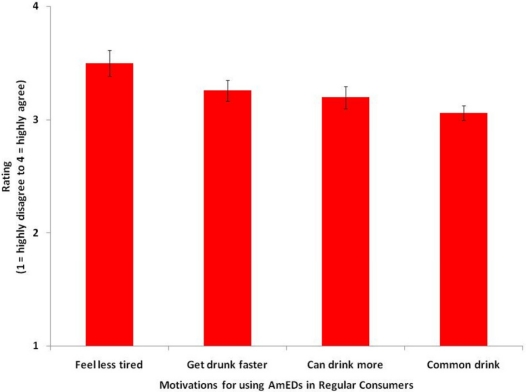
Mean ratings for highest agreement motivations for consuming AmEDs in regular users (n = 66). Standard errors are represented in the figure by the error bars attached to each column.

Table 4 lists the responses from the regular users of AmEDs (n = 66, 9.3% of the whole sample) for possible motivations for using AmEDs. As participants responded on a 4 point Likert scale ranging from 1 (highly disagree) to 4 (highly agree), one-sample t-tests were used to test against the null value of 2.5 to reveal possible motivations for using these beverages. Participants were likely to agree with statements such as: it is a common alcoholic drink, AmEDs allow you to get drunk faster, and I don’t feel as tired when I drink AmEDs. AmED consumers also agreed that AmEDs appeal to underage drinkers (*p* < 0.001). For reference, the same finding was observed for the entire sample (n = 706) with 78% of the sample agreeing or strongly agreeing with the statement that AmEDs appeal to underage drinkers. The top 4 highly rated motivations that were specific to AmEDs are presented in Figure 1.

## 4. Conclusions

The results of this research indicate two major findings. First, the consumption of energy drinks and the consumption of alcohol mixed with energy drinks (AmED) is a relatively common occurrence in college students. The majority of the college students in this sample have tried or are regular consumers of energy drinks (81%). Moreover, 44% of the college students in this sample have tried or are regular consumers of AmEDs. Removing those students who abstain from drinking or who drink alcohol infrequently (44% of the sample), this means that 78% of alcohol users having tried AmEDs or consume them regularly. This finding indicates that AmED consumption has become main stream behavior in college students and is not an isolated phenomenon. Second, the motivations for consuming AmEDs may be leading to more hazardous drinking practices. Motivations for using AmEDs include being able to drink more, to get drunk faster, and to feel less tired while drinking. All participants, regardless of AmED use, reported that they thought that AmEDs are appealing to underage drinkers.

The findings from this study are consistent with previous reports that college students are consuming energy drinks alone and in combination with alcohol, and that this trend is not an isolated development [18,19,25,30]. Previous studies have noted that AmED use, compared to alcohol consumption alone, was associated with greater consumption of quantities alcohol [20] and more deleterious side effects related to drinking, such as being intoxicated, driving after drinking, being injured or requiring medical treatment [19,22]. However, it remained uncertain as to why such outcomes were reported. Recent laboratory evidence suggests that AmEDs lead to enhanced feelings of stimulation while not altering behavioral impairment, compared to alcohol alone [17]. From that lab study, it was plausible to conclude that drinkers might be unaware that AmEDs decrease the sedative properties of alcohol leading the drinker to consume more than intended and resulting in greater risky behavior and more injuries. However, the results of the current study suggest a different interpretation since AmED users state that they are motivated to consume these drinks because they are aware of the sedative-reducing effects of these beverages. They are intentionally choosing them so that they can drink more alcohol than they would be able to otherwise. This suggests that AmEDs may lead to more binge drinking compared to choices of other alcoholic beverages. Moreover, the motives to consume AmEDs are somewhat different than motives to consume energy drinks in isolation. Previous work has established that users of energy drinks are motivated to consume them to increase energy, allay sleepiness, or to boost sports performance [25,27,28]. Prior preliminary research on motivations for consuming AmEDs included wanting to consume the drink while partying and to improve the taste of alcohol [19,25,27,28]. By contrast, our respondents reported being motivated to use AmEDs to alter one’s subjective state when drinking (*i.e.*, feel less tired, get drunk faster). We did not find that our respondents rated improved taste over other forms of alcohol as a major motivation for consuming AmEDs. The role of taste as a motivating factor in the decision to consume AmED beverages warrants further consideration given these different results.

Altering one’s subjective state when drinking may be most problematic on the declining limb of the blood alcohol curve compared to the ascending limb, when the interoceptive cue of sedation is most apparent [31,32]. In the real world when regular alcohol is consumed, it is on the decline of the blood alcohol curve that a drinker feels tired, stops drinking and decides to go home and go to sleep. The AmED user, by contrast, can rely on the stimulant properties of the energy drink to continue drinking more alcohol and for a longer period of time. Disruption of the interoceptive cue of sedation may be a primary reason why AmEDs are riskier than drinking other forms of alcohol [16,17]. For this reason, it may be warranted to consider labeling energy drinks with some form of warning about the risks of combining them with alcohol [21]. In the U.S., such labeling has not occurred.

The present results should be interpreted in light of a few limitations. First, the sample was obtained from one U.S. University, and was a little skewed toward non-Hispanic whites, which limits the generalizability of the results. Although the alcohol consumption patterns reported by the participants were similar to U.S. national norms for college drinking [2–6,14,15], future studies should examine AmED use in college students from a variety of schools, differing in demographic characteristics and from different geographic locations, including outside of the U.S. In addition, market research has revealed that there has been an explosion in the energy drink market over the past decade, with a growth of over 400% from 2003 to 2007. With the worldwide energy drink market estimated at a value of $4.8 billion [24], use rates of energy drinks and AmEDs are probably not stable, but instead likely are increasing. Thus, the current study provides only a snapshot of current consumption rates and future studies should assess use rates of energy drink and AmED use over time to see if AmED use is increasingly in popularity. Finally, the motivations for using these drinks should be replicated and expanded upon, given that the list of possible motivations for AmED use was not exhaustive in this study. Moreover, careful wording of possible motivations is needed to uncover an important distinction between motives for alcohol consumption and AmED consumption. In retrospect, some of our motivations were too general and could have been interpreted by our respondents as answerable in relation to alcohol in general (e.g., the motivation of ‘like the taste’, see Table 4). Given that the motives for alcohol and AmED consumption may differ, carefully worded statements are needed to determine primary motivations for AmED consumption in individuals who consume both types of beverages. In addition, as college students become more familiar with the effects of these beverages, it remains to be seen if they are motivated to use them for the same or different reasons. It is possible that college students may be less motivated to use these drinks over time as they observe more deleterious side effects associated with their use (e.g., seeing peers having accidents or being charged with impaired driving following AmED consumption). Alternatively, the converse could be true as energy drinks gain further popularity and more college students become familiar with the stimulant properties of these beverages and use them as part of their binge drinking activities.

In summary, it appears that alcohol and energy drink co-administration is relatively common among college students and may be contributing to hazardous drinking practices, already a concern with this population that has high rates of binge drinking. The college students who were participants in this study appeared motivated to consume these AmED beverages in a manner consistent with escalating binge drinking (e.g., to suppress sedation, get drunk faster). Moreover, both users and nonusers reported that they thought that AmEDs appeal to underage drinkers. Therefore, it appears that more clinical and research attention should be focused on these alcoholic beverages and how they may be contributing to hazardous drinking practices and future alcohol dependence problems in young people.

## Figures and Tables

**Table 1.** Prevalence estimates of energy drink consumption.

**Characteristics**	**Never tried an energy drink ^a^**		**No recent consumption of energy drink ^b^**		**Recent consumption of energy drinks ^c^**	

	**N**	**%**	**N**	**%**	**N**	**%**
All respondents (n = 706)	131	18.6	318	45.0	257	36.4
**Gender**						
Males (n = 354)	47	13.3	154	43.5	153	43.2
Females (n = 352)	84	23.9	164	46.6	104	29.5
**Age**						
18 (n = 238)	35	14.7	108	45.4	95	39.9
19 (n = 161)	23	14.3	71	44.1	67	41.6
20 (n = 83)	14	16.9	43	51.8	26	31.3
21+ (n = 224)	59	26.3	96	42.9	69	30.8
**Class rank**						
Freshman (n = 412)	61	14.8	188	45.6	163	39.6
Sophomore (n = 122)	26	21.3	52	42.6	44	36.1
Junior (n = 92)	23	25.0	44	47.8	25	27.2
Senior (n = 71)	16	22.5	32	45.1	23	32.4
**Race/ethnicity**						
White (n = 626)	112	17.9	284	45.4	230	36.7
Black or African American (n = 39)	7	17.9	21	53.8	11	28.2
Hispanic or Latino (n = 12)	3	25.0	4	33.3	5	41.7
Asian (n = 10)	4	40.0	2	20.0	4	40.0
Other (n = 19) ^d^	5	26.3	7	36.8	7	36.8

a Never tried an energy drink;

b Have tried an energy drink in the past but no energy drink consumption in the past 2 weeks;

c Have consumed an energy drink in the past 2 weeks;

d Includes American Indian, Alaskan Native, Native Hawaiian, Pacific Islander, and multiracial.

**Table 2.** Prevalence estimates of alcohol drinking.

**Characteristics**	**Nondrinking ^a^**		**Current Drinking With No Binge Drinking ^b^**		**Current drinking with Binge Drinking ^c^**	

	**N**	**%**	**N**	**%**	**N**	**%**
All respondents (n = 706)	307	43.5	138	19.5	261	37.0
**Gender**						
Males (n = 354)	143	40.4	78	22.0	133	37.6
Females (n = 352)	164	46.6	60	17.0	128	36.4
**Age**						
18 (n = 238)	120	50.4	31	13.0	87	36.6
19 (n = 161)	78	48.4	27	16.8	56	34.8
20 (n = 83)	40	48.2	20	24.1	23	27.7
21+ (n = 224)	69	30.8	60	26.8	95	42.4
**Class rank**						
Freshman (n = 412)	199	48.3	63	15.3	150	36.4
Sophomore (n = 122)	53	43.4	22	18.0	47	38.5
Junior (n = 92)	33	35.9	23	25.0	36	39.1
Senior (n = 71)	18	25.4	27	38.0	26	36.6
**Race/ethnicity**						
White (n = 626)	263	42.0	119	19.0	244	39.0
Black or African American (n = 39)	24	61.5	10	25.6	5	12.8
Hispanic or Latino (n = 12)	8	66.7	2	16.7	2	16.7
Asian (n = 10)	3	30.0	5	50.0	2	20.0
Other (n = 19) ^d^	9	47.4	2	10.5	8	42.1
**Greek affiliation (Fraternity/Sorority)**						
No (n = 643)	289	44.9	126	19.6	228	35.5
Yes (n = 63)	18	28.6	12	19.0	33	52.4

a No consumption of alcohol in the past 2 weeks; includes alcohol abstainers;

b Drank at least one drink of alcohol in the past 2 weeks, but did not meet criteria for binge drinking;

c Drank at least one drink of alcohol and drank five or more drinks on one occasion on one or more days during the past 2 weeks for males (drank four or more drinks on one occasion on one or more days during the past 2 weeks for females);

d Includes American Indian, Alaskan Native, Native Hawaiian, Pacific Islander, and multiracial.

**Table 3.** Prevalence estimates of alcohol mixed with energy drinks (AmED) drinking (including survey respondents who did not drink alcohol in the past 2 weeks).

**Characteristics**	**Never tried AmED ^a^**		**Tried AmED but not recently ^b^**		**Recent AmED consumption ^c^**	

	**N**	**%**	**N**	**%**	**N**	**%**
All respondents (n = 706)	395	55.9	245	34.7	66	9.3
**Gender**						
Males (n = 354)	185	52.3	132	37.3	37	10.5
Females (n = 352)	210	59.7	113	32.1	29	8.2
**Age**						
18 (n = 238)	152	63.9	62	26.1	24	10.1
19 (n = 161)	95	59.0	55	34.2	11	6.8
20 (n = 83)	44	53.0	33	39.8	6	7.2
21+ (n = 224)	104	46.4	95	42.4	25	11.2
**Class rank**						
Freshman (n = 412)	243	59.0	133	32.3	36	8.7
Sophomore (n = 122)	62	50.8	50	41.0	10	8.2
Junior (n = 92)	52	56.5	30	32.6	10	10.9
Senior (n = 71)	33	46.5	30	42.3	8	11.3
**Race/ethnicity**						
White (n = 626)	347	55.4	219	35.0	60	9.6
Black or African American (n = 39)	26	66.7	10	25.6	3	7.7
Hispanic or Latino (n = 12)	6	50.0	5	41.7	1	8.3
Asian (n = 10)	7	70.0	3	30.0	0	0.0
Other (n = 19) ^d^	9	47.4	8	42.1	2	10.5
**Greek affiliation (Fraternity/Sorority)**						
No (n = 643)	360	56.0	225	35.0	58	9.0
Yes (n = 63)	35	55.6	20	31.7	8	12.7
**Binge drinker ^e^**						
No (n = 445)	321	72.1	108	24.3	16	3.6
Yes (n = 261)	74	28.4	137	52.5	50	19.2

a Never tried alcohol mixed with energy drinks (AmED); includes alcohol abstainers;

b Have tried an AmED in the past but no AmED consumption in the past 2 weeks;

c Have consumed at least one AmED in the past 2 weeks;

d Includes American Indian, Alaskan Native, Native Hawaiian, Pacific Islander, and multiracial;

e Binge drinker is defined as having drank five or more alcoholic drinks on one occasion on one or more days during the past 2 weeks for males (drank 4 or more drinks on one occasion on one or more days during the past 2 weeks for females).

**Table 4.** Motivations for using AmEDs in regular users (n = 66). Responses were made on a 4 point Likert scale ranging from 1 (highly disagree) to 4 (highly agree). Significance refers to a one-sample *t* test (2-tailed) testing against a 2.5 null value.

**Motivation for using AmED**	**Mean**	**S.D.**	**Sign.**
*Do you agree or disagree with the following regarding AmEDs:*			
It is a common drink	3.06	0.52	0.000
AmEDs help you hold your liquor better	2.08	0.62	0.000
AmEDs are the same as other mixed drinks	2.36	0.67	*ns*
AmEDs taste better than other alcoholic drinks	2.50	0.81	*ns*
I can drink more if I drink AmEDs	3.20	0.79	0.000
AmEDs allow you to get drunk faster	3.26	0.75	0.000
I don’t feel as tired when I drink AmEDs	3.50	0.92	0.000
*How important is the following reasons for you to drink AmEDs:*			
To get away	2.41	0.78	*ns*
To relax	2.56	0.70	*ns*
To socialize	2.95	0.75	0.000
To get drunk	2.82	0.86	0.004
To celebrate	3.00	0.89	0.000
To have something to do	2.48	0.71	*ns*
To get work done	2.23	0.60	0.000
Like the taste	3.02	0.83	0.000
Reward myself	2.53	0.81	*ns*
To fit in	2.36	0.69	*ns*
To feel more appealing to the opposite sex	2.47	0.75	*ns*
Because everyone else is doing it	2.42	0.75	*ns*
Because it’s cheap	2.38	0.67	*ns*
